# Supplementary material for: Causal Relationship between Aging and Anorexia Nervosa: A White-Matter-Microstructure-Mediated Mendelian Randomization Analysis
Source: Biomedicines. 2024 Aug 16;12(8):1874. doi: 10.3390/biomedicines12081874 (PMC11351342; doi:10.3390/biomedicines12081874)
Supplement: Supplementary file 1 [file biomedicines-12-01874-s001.zip › Supplementary Figure/Figure S4/Singe SNP plot of MR analysis for TL to ubm-b-1831.pdf]

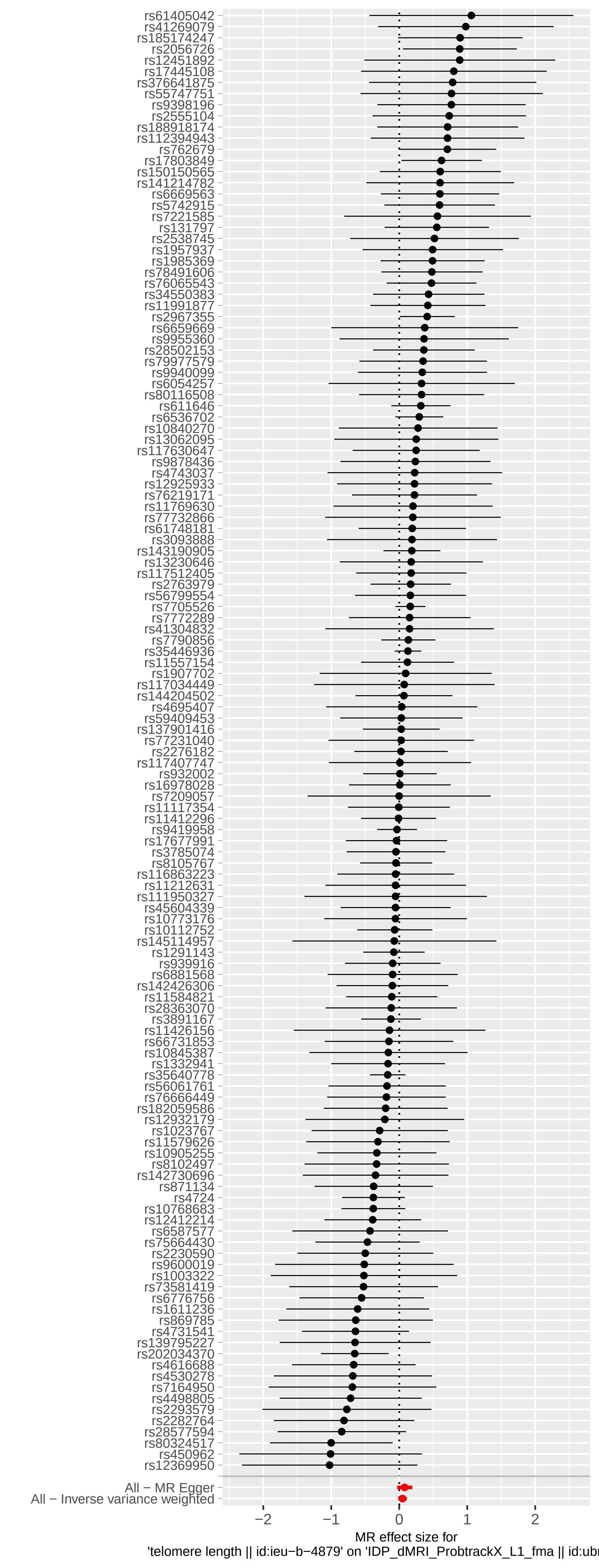
rs61405042
rs41269079
rs185174247
rs2056726
rs12451892
rs17445108
rs376641875
rs55747751
rs9398196
rs2555104
rs188918174
rs112394943
rs762679
rs17803849
rs150150565
rs141214782
rs6669563
rs5742915
rs7221585
rs131797
rs2538745
rs1957937
rs1985369
rs78491606
rs76065543
rs34550383
rs11991877
rs2967355
rs6659669
rs9955360
rs28502153
rs79977579
rs9940099
rs6054257
rs80116508
rs611646
rs6536702
rs10840270
rs13062095
rs117630647
rs9878436
rs4743037
rs12925933
rs76219171
rs11769630
rs77732866
rs61748181
rs3093888
rs143190905
rs13230646
rs117512405
rs2763979
rs56799554
rs7705526
rs7772289
rs41304832
rs7790856
rs35446936
rs11557154
rs1907702
rs117034449
rs144204502
rs4695407
rs59409453
rs137901416
rs77231040
rs2276182
rs117407747
rs932002
rs16978028
rs7209057
rs11117354
rs11412296
rs9419958
rs17677991
rs3785074
rs8105767
rs116863223
rs11212631
rs111950327
rs45604339
rs10773176
rs10112752
rs145114957
rs1291143
rs939916
rs6881568
rs142426306
rs11584821
rs28363070
rs3891167
rs11426156
rs66731853
rs10845387
rs1332941
rs35640778
rs56061761
rs76666449
rs182059586
rs12932179
rs1023767
rs11579626
rs10905255
rs8102497
rs142730696
rs871134
rs4724
rs10768683
rs12412214
rs6587577
rs75664430
rs2230590
rs9600019
rs1003322
rs73581419
rs6776756
rs1611236
rs869785
rs4731541
rs139795227
rs202034370
rs4616688
rs4530278
rs7164950
rs4498805
rs2293579
rs2282764
rs28577594
rs80324517
rs450962
rs12369950
All − MR Egger
All − Inverse variance weighted
−2
−1
0
1
2
MR effect size for
'telomere length || id:ieu−b−4879' on 'IDP_dMRI_ProbtrackX_L1_fma || id:ub
